# Genetic mutation spectrum of pantothenate kinase-associated neurodegeneration expanded by breakpoint sequencing in pantothenate kinase 2 gene

**DOI:** 10.1186/s13023-022-02251-7

**Published:** 2022-03-04

**Authors:** Dahae Yang, Sanghyun Cho, Sung Im Cho, Manjin Kim, Moon-Woo Seong, Sung Sup Park

**Affiliations:** 1 Department of Laboratory Medicine, Kosin Gospel University Hospital, Busan, Korea; 2 Department of Laboratory Medicine, Seoul National University Hospital, 101 Daehak-ro, Jongno-gu, Seoul, 03080 Korea; 3 Biomedical Research Institute, Seoul National University Hospital, Seoul, Korea

## Abstract

**Background:**

Neurodegeneration with brain iron accumulation describes a group of rare heterogeneous progressive neurodegenerative disorders characterized by excessive iron accumulation in the basal ganglia region. Pantothenate kinase-associated neurodegeneration (PKAN) is a major form of this disease.

**Results:**

A total of 7 unrelated patients were diagnosed with PKAN in a single tertiary center from August 2009 to February 2018. Ten variants in *PANK2* including three novel sequence variants and one large exonic deletion were detected. Sequencing of the breakpoint was performed to predict the mechanism of large deletion and AluSx3 and AluSz6 were found with approximately 97.3% sequence homology.

**Conclusion:**

The findings support the disease-causing role of *PANK2* and indicate the possibility that exonic deletion of *PANK2* found in PKAN is mediated through *Alu*-mediated homologous recombination.

## Introduction

Neurodegeneration with brain iron accumulation (NBIA) describes a group of rare heterogeneous progressive neurodegenerative disorders characterized by excessive iron accumulation in the basal ganglia region [1]. Pantothenate kinase-associated neurodegeneration (PKAN) accounts for 30–50% of NBIA cases with prominent extrapyramidal dysfunction, with estimated prevalence of one to two per million live births worldwide [2–4]. Diagnosis of PKAN usually consists of the clinical, radiological, and genetic background of the patient. Based on age, disease progression rate, and clinical symptoms, PKAN has two main presentations: a classic variant with severe early-onset and an atypical variant with more variable phenotype with later age at onset [5, 6].

Pantothenate kinase 2 (*PANK2*)*,* which is considered the causative gene of PKAN, is located on chromosome 20p13 and encodes a 50.5 kDa mitochondrial enzyme called pantothenic acid kinase 2 (PanK-II), which is involved in the first regulatory step of coenzyme A biosynthesis [6]. Although the exact pathology of PKAN has not been established, debilitating variants in the *PANK2* gene assumedly enable sufficient conversion of pantothenate to 4′-phosphopantothenate, leading to mitochondrial cysteine accumulation [7, 8]. Cysteine, which effectively binds iron, is thought to undergo rapid auto‐oxidation, resulting in cytotoxic free radical production, which triggers cell damage [9]. Accordingly, most patients with *PANK2* variant are known to exhibit a specific radiologic pattern of hyperintensity within the hypointense medial globus pallidus, called the eye-of-the-tiger sign, on T2-weighted MR imaging [2].

In this study, the molecular spectrum was expanded and the possible underlying disease causing the mechanism of PKAN investigated.

## Materials and methods

1. Subjects

**Fig. 1:**
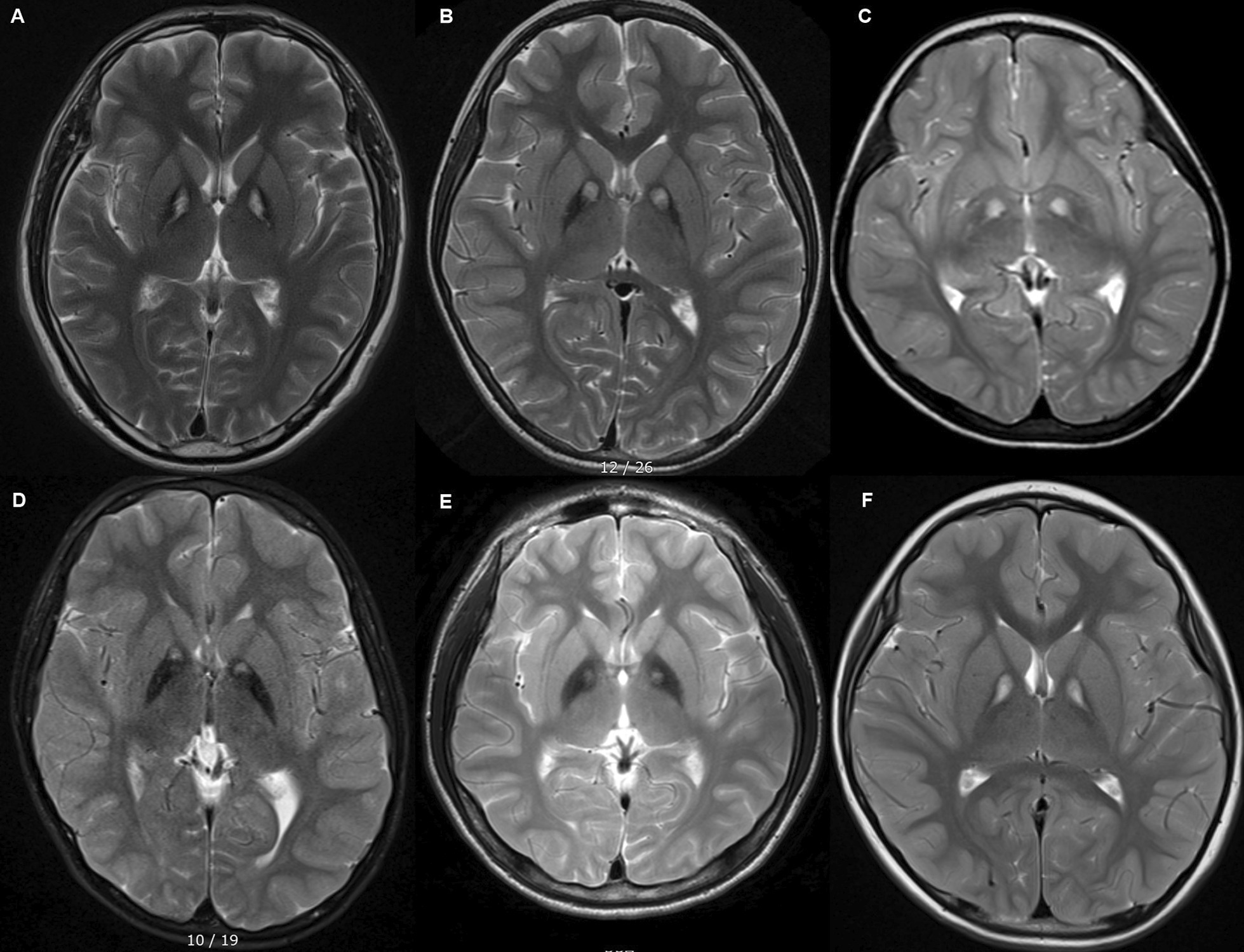
MRI T2 image of **A** P1, **B** P3, **C** P4, **D** P5, **E** P6, **F** P7 showing symmetrical central hyperintensity surrounded by hypointensity in the globus pallidus giving “eye’s of tiger sign” appearance

From August 2009 to February 2018, patients with suspected PKAN referred to Seoul National University Hospital (SNUH) were enrolled in this study (Table 1, Fig. 1). The Institutional Review Board of SNUH approved the study.

2. Sanger sequencing

**Table 1:** Clinical characteristics of patients enrolled in this study

Patient	P1	P3	P4	P5	P6	P7
Gender	M	F	F	M	M	M
Age of onset (yr)	30	8	14 months	2	12	27
Presenting symptoms	Right hand dystonia	Decreased visual acuity	Frequent fall	Development delay	Gait disturbance	Dystonia
Age at diagnosis (yr)	37	10	3	9	15	47
Dystonia	Limb, trunk	Limbs, trunk	Upper limbs	Neck, limbs	Generalized dystonia, opisthotonus	General dystonia, blepharospasm
Bulbar symptoms	Dysarthria	Dysphagia	Dysarthria, dysphagia	Dysarthria		Dysarthria, dysphagia
Gait/ posture problems	( −)	Dystonic posturing, unsteady	Ataxic gait, tip toeing	Dystonic gait	Foot drop, tip toeing	Slow, reduced arm swing, unsteady
Others	( −)	Retinopathy, respiratory difficulty	( −)	autistic behavior, seizure	cognitive decline	insomnia, parkinsonism (bradykinesia, resting tremor, postural instability)
Family history	Denied	Denied	AR	AR	Denied	Sister: NE
Eyes of tiger sign	( +)	( +)	( +)	( +)	( +)	( +)

*NE* not examined

*Clinical data of P2 is unavailable

Genomic DNA was extracted from each patient's EDTA peripheral blood using Gentra Puregene DNA isolation kits (Gentra Systems, Inc., Minneapolis, MN, USA) according to the manufacturer's protocol. The extracted DNA was measured at 260 nm using a Nanodrop-2000 (Nanodrop Technologies, Wilmington, DE, USA) and DNA quality was confirmed based on 2% agarose gel electrophoresis. PCR was performed using in-house primers in the region containing the coding exons of the *PANK2* gene and the flanking region of the intron. PCR products were electrophoresed on 2% agarose gel for verification by comparison with expected product size. The PCR product was purified using ExoSAP-IT (USB, Cleveland, OH, USA) at 37 °C for 30 min and at 80° C for 15 min. Twenty-five cycles of sequencing reaction (95 °C for 10 s, 50 °C for 5 s, 60 °C for 4 min) were performed with BigDye Terminator v3.1 Cycle Sequencing Kits (Applied Biosystems, Foster, CA, USA). Dye was purified with a SAM/X-Terminator solution and sequenced using an ABI 3730 Genetic Analyzer (Applied Biosystems, Inc., Foster City, CA, USA). The obtained results were compared with the reference sequence (GeneBank ID: NG_008131.3) using the Seqscape software v5.0 (Applied Biosystems).

3. Multiplex ligation-dependent probe amplification (MLPA)

Genomic rearrangement screening was performed using SALSA P120-B2 *PANK2*/*PLA2G6* Kits (MRC Holland, Amsterdam, The Netherlands). First, 200 ng of genomic DNA was mixed with RNase, denatured at 98 °C, and hybridized with probe mix at 60 °C for 16 h. Ligation was performed at 54 °C for 15 min with the Ligase-65 mix, followed by multiplex PCR using universal primers and SALSA polymerase for 35 cycles (95 °C denaturation for 30 s, 60 °C for 30 s, 72° C for 1 min). Fragment analysis was performed on an ABI 3130 XL capillary sequencer (Applied Biosystems) with 1 µL of final PCR product, 0.2 µL of GeneScan-500ROX Size Standard (Applied Biosystems), and 8.8 µL of HiDi Formamide (Applied Biosystems). Results were analyzed based on population normalization of peak height using Genemarker software v1.51 (SoftGenetics, State College, PA, USA).

4. Deletion breakpoint evaluation

A further experiment was conducted to identify the deletion breakpoint using the genomic DNA of an exon 3–4 deletion sample confirmed based on multiplex ligation-dependent probe amplification (MLPA). In-house primers were designed to examine the exon and intron regions of the 3–4 deletion areas, and long-PCR was performed using the Takara LA PCR System (Takara BIO INC, Shiga, Japan). Long-range PCR products were electrophoresed on 0.7% agarose gel at 90 V for 5 h to confirm PCR results.

Sanger sequencing was performed and the results compared with the reference sequence to determine the exact origin of fruiting and deletion size. To predict the mechanism of large deletion, RepeatMasker (http://www.repeatmasker.org/cgi-bin/WEBRepeatMasker/) was used to identify the type of repetitive elements and Clustal W (http: //www.genome.jp/tools-bin/clustalw/) software to perform multiple sequence alignments.

## Results

1. Characteristics of variants spectrum in *PANK2*

A total of 10 variants were detected in 7 patients (Table 2). Missense variants (60%) were most common, followed by small deletion (20%), duplication (10%), and exonic deletion (10%). Six of the 10 identified variants were previously reported (five missense and one small deletion), and four (40%) variants were novel. According to the American College of Medical Genetics and Genomics (ACMG) 2015 guidelines, c.1210_1214dupAATTA, c.823_824delCT were considered likely pathogenic (LP) and c.1315G > T was considered a variant of uncertain significance (VUS). Among the variants, c.1319G > C, p.Arg440Pro was the most frequent and identified in 42.6% (3/7) of patients.

2. Discovery and verification of a large deletion

**Table 2:** *PANK2* gene variants detected using Sanger sequencing and MLPA

Patient	Nucleotide change*	Amino acid change	Zygosity	ACMG	References
P1	c.1133A > G	p.Asp378Gly	Heterozygote	LP	[22]
	c.1273_1275delCTT	p.Leu425del	Heterozygote	LP	[23]
P2	c.1133A > G	p.Asp378Gly	Heterozygote	LP	[22]
	c.1319G > C	p.Arg440Pro	Heterozygote	LP	[11]
P3	c.852 T > G	p.Phe284Leu	Heterozygote	VUS	[24]
	c.1319G > C	p.Arg440Pro	Heterozygote	LP	[11]
P4	c.1210_1214dupAATTA	p.Tyr405*	Heterozygote	LP	This study
	c.1319G > C	p.Arg440Pro	Heterozygote	LP	[11]
P5	c.1273_1275delCTT	p.Leu425del	Heterozygote	LP	[23]
	c.1676C > G	p.Ala559Gly	Heterozygote	VUS	[24]
P6	c.1607A > G	p.Tyr536Cys	Heterozygote	LP	[24]
	Exon 3–4 del		Heterozygote		This study
P7	c.823_824delCT	p.Leu275Valfs*16	Heterozygote	LP	This study
	c.1312G > T	p.Ala438Ser	Heterozygote	VUS	This study

*Numbering is according to cDNA sequences (GenBank: NM_153638.2)

The A of the ATG of the initiator methionine codon is denoted as nucleotide 1

MLPA, multiplex ligation-dependent probe amplification; ACMG, American College of Medical Genetics and Genomics; LP, likely pathogenic; VUS, variant of uncertain significance

**Fig. 2:**
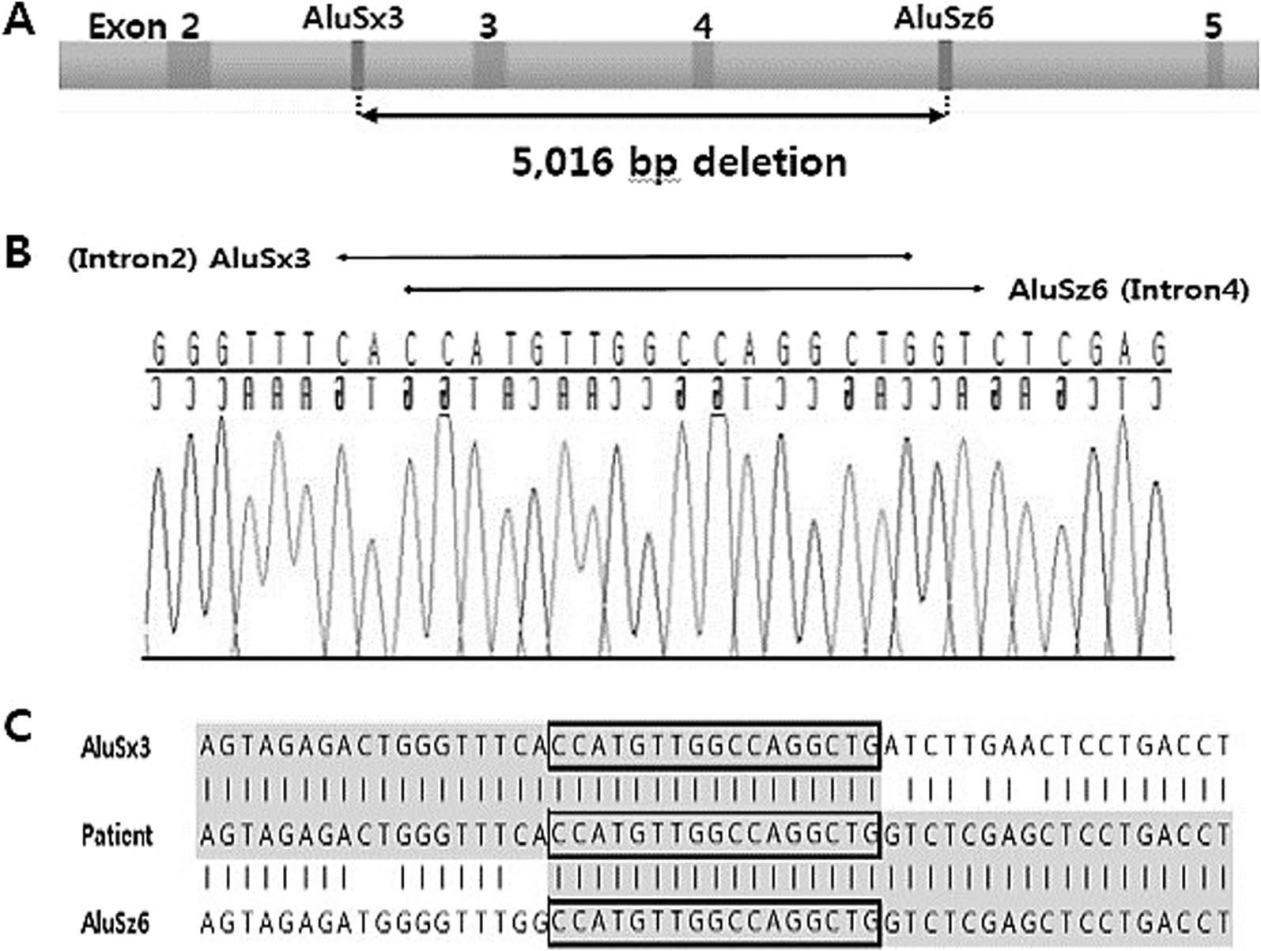
Breakpoint mapping and characterization of the *PANK2* exon 3–4 deletion. **A** Schematic representation of 5016 bp deletion breakpoints. **B** Sequence analysis showing breakpoints of the exon 3–4 large deletion. Breakpoints of this deletion are inside *Alu* repeats indicating *Alu*-mediated homologous recombination. **C** Sequence alignment at deletion breakpoints. Boxes indicate the *Alu*-sequence homology

Based on MLPA results, heterozygous exon 3–4 deletion was confirmed in patient 6 (P6; Table 2). To characterize the deletion breakpoint, a pair of in-house primers was constructed for sequencing. The product size of the alleles with deletion was approximately 270 bp and the reference strand was approximately 5300 bp. Sequence analysis of the 270 bp fragment confirmed the exon 3–4 deletion was a 5,016 bp deletion in the range of g.25,954–30,969 with reference genome sequence NG_008131.3 (c.982-785_1413-2120del with the reference DNA sequence NM_153638.3). The sequence of the breakpoint was found to be AluSx3 and AluSz6 and sequence homology was approximately 97.3% (Fig. 2).

## Discussion

PKAN is a progressive neurodegenerative disorder affecting movement, cognition, and behavior, and effective treatment does not exist [10]. Pantothenate kinase (PanK), a regulator in CoA biosynthesis, has three distinct types [7]. Among them, PanK-II is the sole mitochondria-targeted human PanK involved in various metabolic cycles with four human isoforms (PanK1α, PanK1β, PanK2, and PanK3) [11, 12]. The PanK2 encoded by the *PANK2* gene is abundant in human neuronal tissues. The majority of the *PANK2* variants known associated with PKAN result from little or no catalytic activity of PanK2 enzyme.

PKAN is a very rare disease, and only approximately 180 variants have been reported worldwide [13]. Most variants exist in the core catalytic region; in our study, all variants were within the range of 212–570 amino acids. Three hotspot variants (D378G, G521R, and T528M) in the *PANK2* gene were previously reported [14], however, R440P was most common in the present study. Previously, Lim et al. [11] suggested the R440P variant, which occurred in 38% of PKAN patients, as a Korean-specific prevalent variant. In our case, R440P was reported with similar prevalence to the previous report, supporting the idea of a racial characteristic. Further research is needed to investigate the possibility of a founder effect on R440P.

In the present study, four novel variants, including three sequence variants and one large structural variant, in apparently unrelated families of PKAN were described.

The discovery of large deletions spanning exon 3–4 is noteworthy. Large deletions in PKAN have been reported in only a few studies and large deletions across exons are particularly rare [2, 11, 12]. In only one previous report, a large deletion was assumed to have occurred via two highly homologous sequences (92%) encompassing the breakpoint, however, the authors did not perform further evaluation [12].

To predict the mechanism of this deletion, the presence of repetitive elements in the vicinity of deletion breakpoints was examined for possible associations. The elements identified in the present study were AluSx3 and AluSz6. *Alu* elements are a family of short, interspersed elements that are RNA polymerase III transcribed retrotransposons which replicate via long interspersed element-encoded proteins and play an important role in genetic diversity [15, 16]. More than one million *Alu* elements comprise roughly 10% of the human genome [17]. Although *Alu* repeats have no known biological function, *Alu*/*Alu* recombination has been reported to generate approximately 0.5% of human genetic diseases, particularly structural variations [18, 19]. Recombination between these *Alu* elements tends to occur through highly homologous *Alu* sequences [20]. The concordance between the two *Alu* sequences found in the present study reached 97.3%. Consequently, we infer the exon 3–4 deletion identified in this study was induced due to homologous recombination by AluSx3 and AluSz6.

Although in previous reports missense variants were suggested the main cause of early and late form PKAN [7], the occurrence of these diseases caused by truncated variants has consistently been reported [21]. Furthermore, null alleles were recently reported to be associated with more aggressive progression [13]. Therefore, finding the null variant of PKAN and understanding the mechanism of its occurrence is important for diagnosing and predicting the severity of the disease.

## Conclusion

The present study results support the disease-causing effects of *PANK2* in PKAN and are significant as the first report indicating that Alu-mediated homologous recombination is responsible for large deletion underlying PKAN. A large deletion in the *PANK2* gene should not be overlooked in cases in which molecular diagnosis cannot be achieved through routine workup.

## Data Availability

Data reasonably requested can be provided by the corresponding author after discussion with the Institutional Review Board.
